# Phylogenetic analysis of feline infectious peritonitis virus, feline enteric coronavirus, and severe acute respiratory syndrome coronavirus 2 of cats in Surabaya, Indonesia

**DOI:** 10.14202/vetworld.2023.76-81

**Published:** 2023-01-11

**Authors:** Eduardus Bimo Aksono, Kania Rifa Iradatya, Teguh Hari Sucipto, Nur Syamsiatul Fajar, Wiwik Misaco Yuniarti

**Affiliations:** 1 Department of Veterinary Medicine, Faculty of Veterinary Medicine, Universitas Airlangga, Surabaya 60115, Indonesia; 2 Natural Science and Engineering Institute, Universitas Airlangga, Surabaya 60115, Indonesia; 3 Institute of Tropical Disease, Universitas Airlangga, Surabaya 60115, Indonesia

**Keywords:** cats, feline coronavirus, feline infectious peritonitis virus, phylogenetic, severe acute respiratory syndrome coronavirus 2, Surabaya

## Abstract

**Background and Aim:**

Questions about the origin of coronavirus and its introduction to human beings have persisted. The detection of a variety of coronavirus related to severe acute respiratory syndrome coronavirus 2 (SARS-CoV-2) in bats and pangolins led to the widespread belief that SARS-CoV-2 originated from wild ani­mals and was introduced to humans through an inter­mediate animal. Thus, coronaviruses from animals, especially those in close contact with humans, have attracted particular attention. This study aimed to phylogenetically analyze feline enteric coronavirus (FECV), feline infectious peritonitis virus (FIPV), and SARS-CoV-2 found in cats in Surabaya amid the COVID-19 pandemic. The results will provide a basis for developing basic preventive and pet healthcare strategies.

**Materials and Methods:**

Samples were collected on physical examinations of domestic and Persian cats (males and females) from March 2020 to March 2022. Samples were collected if there were clinical signs of FECV and FIP based on a veterinarian’s diagnosis in several clinics in Surabaya. Laboratory examinations in this study were performed by reverse-transcription-polymerase chain reaction (RT-PCR) with primers for conserved regions of FIP and FECV, DNA sequencing was performed with Applied Biosystem Genetic Analyzer protocol, homology analysis was performed using Basic Local Alignment Search Tool NCBI, phylogenetic analysis was carried out with BioEdit 7.2 software, and sequences were compared with references from GenBank.

**Results:**

Samples were collected from ten cats showing clinical signs of FECV and FIP, based on a veterinarian’s diagnosis. On RT-PCR examinations performed with specifically designed primers for detecting FIPV in blood, peritoneal fluid, and feces, only one sample showed positivity for FIPV (1/10), namely, a peritoneal sample from a domestic cat in Surabaya. Homology analysis of the FIPV Surabaya isolate showed 98% similarity with FECV and FIPV reported in GenBank (MT444152 and DQ010921, respectively). In phylogenetic analysis, the FIPV Surabaya isolate was clustered together with SARS-CoV-2 of Clade A (MT198653) from Spain, SARS-CoV-2 Clade A (MT192765) from the USA, SARS-CoV-2 Clade D (039888) from the USA, and SARS-CoV-2 Clade F (MT020781) from Finland.

**Conclusion:**

This study revealed a relationship between the SARS-CoV-2 viruses that infect humans and cats (FECV), which is an important finding for those keeping cats at home. However, this finding requires further comprehensive support from laboratory studies.

## Introduction

The coronavirus disease 2019 (COVID-19) pandemic has now lasted for 3 years, but until now there are still reported cases every day. Besides being able to infect humans, this virus can also infect pets such as cats, for example, Feline infectious peritonitis virus (FIPV). This virus causes a fatal feline disease and is a variant of feline enteric coronavirus (FECV). Both FECV-related disease and severe acute respiratory syndrome coronavirus 2 (SARS-CoV-2) are diseases caused by coronavirus, which have certain similarities. Both are highly transmissible, for which similar infection preventive measures have been instigated by isolating infected patients. Some anti-inflammatory compounds and antiviruses also show similar efficacy against them. However, some differences have also been identified in terms of virus biological character, targeted cells, pathogenesis, and clinical signs. The similarities and differences between FECV and SARS-CoV-2 can be used to obtain a deeper understanding of cell biological aspects andhost–virus interaction, which could aid the development of preventive strategies and effective therapies for FIP and COVID-19. It is highly possible that FECV and SARS-CoV-2 will interact further in the future due to humans and their pet cats living in close proximity [1]. Furthermore, amid the COVID-19 pandemic, cat owners are worried about their pets being a source of infection, considering that the number of FIP cases has also increased.

Feline coronavirus and SARS-CoV-2 are single-stranded RNA viruses belonging to the order Nidovirales and family Coronaviridae. Feline coronavirus was originally classified into the genus *Alphacoronavirus* and SARS-CoV-2 into the genus *Betacoronavirus*. Feline coronavirus has a different genomic structure than SARS-CoV-2, with 5¢-leader-UTR-replicase-S (Spike)-E (Envelope)-M (Membrane)-N (Nucleocapsid)-3¢UTR-poly(A) tail and genes interspersed within the structural genes at the 3¢-end of the genome.

Practitioners of veterinary medicine have long been familiar with coronavirus because its species variants (CoV) can cause various health problems in wild animals such as bats and birds, as well as in domestic animals such as pigs, cows, cats, and dogs [1–4]. An infectious peritonitis disease found in cat populations, especially in those under 2 years old, is FECV disease [5]. This disease shows non-specific clinical signs and laboratory changes, making its diagnosis a challenge for vets and researchers. Etiologically, feline coronavirus (FCoV) has two pathotypes. The first is the FECV pathotype, which is commonly found and highly contagious. It has a transmission rate of almost 100%, although most cats are asymptomatic or exhibit only mild diarrhea. The major transmission route is fecal-oral, through FECV-contaminated feces or food [3, 6–8]. The second pathotype is FIPV. This pathotype occurs not through contaminated food or fecal transmission but from mutations of some avirulent FECV that is generally non-infectious. However, such mutation leads to a fatal disease in cat populations, FIP [8, 9]. The connection between FECV and FIPV has been debated. From genetic and experimental evidence in animals, it has been shown that FIPV occurs as a mutated form of FECV in the continuously exposed intestinal tract of cats. However, the gene whose mutation is associated with the shift from FECV to FIPV remains unknown.

In Surabaya, Indonesia, a suspected case of FIP in a Turkish Angora was reported, which was diagnosed based on clinical, anatomical pathology, and histopathological signs [10]. Cats are rarely infected by FIPV through direct contact. Instead, this deadly disease is generally derived from FECV mutating into FIPV. One clinical sign of this disease is digestive problems in cats. Feline infectious peritonitis infects Persian and domestic cats. In terms of the survival of cats infected by FIP, this can range from 1 week to 1 year. Unfortunately, most diagnostic kits developed to date cannot differentiate between FECV and FIPV-related disease, making it difficult for vets to diagnose FIP, especially in cats without effusion from the body cavity. Against this background, it is important to perform research to aid the definitive antemortem diagnosis of FIPV in infected cat populations [8]. Chang *et al*. [11] and Brown *et al*. [12] suggested that five amino acid residues in the matrix (M) protein can be used to differentiate virulent FIPV from avirulent FECV; meanwhile, Felten and Hartmann [8] reported that a single-nucleotide polymorphism in the S gene is found only in cats with FIP, but not in healthy cats with FECV.

Questions about the origin of coronavirus and its introduction to human beings have persisted. The detection of a variety of coronavirus related to SARS-CoV-2 in bats and pangolins led to the widespread belief that SARS-CoV-2 originated from wild animals and was introduced to humans through an intermediate animal. Thus, coronaviruses from animals, especially those in close contact with humans, have attracted particular attention.

This study aimed to phylogenetically analyze FECV, FIPV, and SARS-CoV-2 found in cats in Surabaya amid the COVID-19 pandemic. The results will provide a basis for developing basic preventive and pet healthcare strategies.

## Materials and Methods

### Ethical approval

This study was approved for animal experimentation by the Commission of Research Ethics of the Faculty of Veterinary Medicine Universitas Airlangga (No. 1. KE. 014.01.2020).

### Study period and location

This study was conducted from the beginning of the COVID-19 pandemic, from March 2020 to March 2022, using samples from 10 domestic or Persian cats, male or female, of any age. All the cats showed clinical signs of FECV and FIP based on a veterinarian’s diagnosis in several clinics in Surabaya, Indonesia. The polymerase chain reaction (PCR) examinations, sequencing, homology, and phylogenetic analysis were performed at the Institute of Tropical Disease Universitas Airlangga and the Institute of Natural Sciences and Engineering Surabaya.

### Sampling

Three different sample types were used in this study (blood, n = 10; peritoneal fluid, n = 10; feces, n = 10). A primer pair for a conserved region was specifically designed to detect FIPV and FECV (F2: 5¢-TCTTGCTAACTGGAACTTCAGCTGG-3¢, R2: 5¢-TGACGCGTTGTCCCTGTGTG-3¢).

A total of 1 or 2 mL of cat blood was collected and put into a tube with ethylenediaminetetraacetic acid. The peritoneal fluid samples were stored in a sterile tube with a screw cap. Meanwhile, 400 μL of PBS was added to 25 mg of feces and mixed with a vortex mixer. It was then centrifuged at 15,300× *g* for 3 min. The obtained supernatant was then used for the extraction of RNA using the QIAamp RNA Viral Mini Kit (Qiagen, Hilden, Germany), in accordance with the manufacturer’s procedure.

### Reverse transcription-polymerase chain reaction (RT-PCR) and DNA visualization

The reverse-transcription reaction followed the protocols of ReverTra Ace quantitative PCR RT Master kit with gDNA Remover from Toyobo (Germany). A total of 2 μL of DN Master was supplemented with gDNA Remover, 1 μL of Random Primer, 2 μL of nuclease-free water, 2 μL of 5× reverse transcriptase Master Mix II, and 3 μL of RNA samples and mixed in a reaction volume of 10 μL. This mixture was then incubated at 65°C for 1 min, at 37°C for 15 min, at 42°C for 30 min, and at 98°C for 5 min.

After the RT reaction, amplification proceeded with a mixture of 12.5 μL of 2× PCR Master Mix (Intron), l μL of F2 (10-nmol forward primer), 1 μL of R2 (10-nmol reverse primer), 2.5 μL of nuclease-free water, and 3 μL of cDNA sample.

The polymerase chain reaction was conducted with a Bioer thermal cycler. The protocol consisted of pre-denaturation at 94°C for 5 min; 40 cycles of denaturation at 94°C for 30 s, annealing at 58°C for 30 s, and extension at 72°C for 30 s; and final extension at 72°C for 5 min.

The output from PCR was subjected to electrophoresis with a gel containing 2% agarose and TBE1× (Promega, Madison, USA), as well as RedSafe (iNtRON Biotechnology, Gyeonggi-do, Republic of Korea) as an agarose gel dye. Electrophoresis was performed with a Mupid-eXu apparatus (Advance) at 100 V for 35 min. NexMark 100 bp DNA markers were used as molecular weight markers.

### DNA Sequencing and Phylogenetic Analysis

Peritoneal fluid showing a positive PCR result was then purified using QIAquick DNA Purification Kit (Qiagen). Sequencing was then performed in accordance with the Applied Biosystem Genetic Analyzer protocol. Homology analysis was conducted with a positive sample at https://blast.ncbi.nlm.nih.gov/Blast.cgi. Phylogenetic analysis of FIPV Surabaya isolate was performed using reference sequences from GenBank: FCoV (MT444152), FIPV (DQ010921), and SARS-CoV-2, including MT135041 (China), MT135042 (China), MT135044 (China), MN994467 (USA), MT152824 (USA), MT163717 (USA), MT198652 (Spain), MT198653 (Spain), MT039888 (USA), MT020781 (Finland), MT192765 (USA), LC529905 (Japan), LC529905 (Japan), MT066156 (Italy), MT007544 (Australia), and MT039890 (Republic of Korea).

## Results

**Figure-1:**
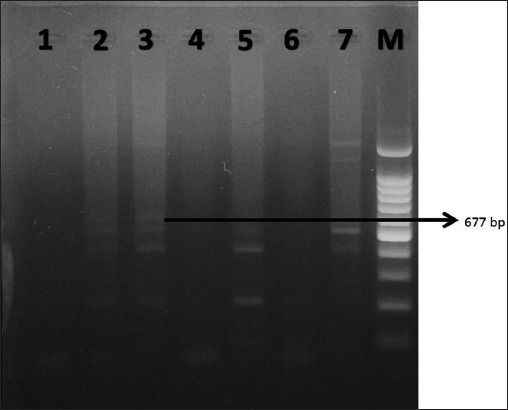
Reverse transcription-polymerase chain reaction electrophoresis result for feline infectious peritonitis virus. M-Marker DNA 100 bp. 1-Control negative; 2-Peritoneal liquid (negative); 3-Peritoneal liquid (positive); 4-Blood (negative); 5-Blood (negative); 6-Feces (negative); and 7-Feces (negative).

Table-1 shows the samples from 10 cats suspected of having FECV and FIP. In terms of their breeds, there were six domestic cats and four Persian cats. In terms of the FIP types, seven cats had the FIP wet-type and three had the FIP Dry-type. Regarding their ages, six were kittens (0–6 months), one was a mature cat (7–10 years), two were junior cats (7 months –2 years), and one was a senior cat (11–14 years). Moreover, seven cats were male and three were female. In the physical examinations, all cats showed clinical signs of FECV and FIP, including diarrhea, anorexia, pale mucous membranes, pyrexia, dyspnea, abdominal distension, and weight loss. Reverse transcription-polymerase chain reaction (RT-PCR) examinations of blood, peritoneal liquid, and fecal samples with specifically designed primers for conserved regions of FIPV and FECV revealed positivity for FIPV (1/10) in one sample, in the Figure-1 shown DNA band at 677 bp, which was from peritoneal fluid from a domestic cat. This low isolation rate was believed to be associated with poor storage conditions for the samples during transport.

**Table-1:** The clinical sign and RT-PCR examinations from samples of domestic and Persian cats for FIPV Surabaya isolate amid the COVID-19 pandemic.

Case number	Clinical signs	Specimen	RT-PCR
Domestic 1: Male, Kitten, Wet-type	Diarrhea, anorexia, pale mucous Membranes, pyrexia, dyspnea Abdominal distension, weight loss	Blood	Negative
		Peritoneal liquid	Negative
		Feces	Negative
Domestic 2: Female, Mature, Wet-type	Diarrhea, anorexia, pale mucous Membranes, pyrexia, dyspnea Abdominal distension, weight loss	Blood	Negative
		Peritoneal liquid	Negative
		Feces	Negative
Domestic 3: Male, Kitten, Wet-type	Diarrhea, anorexia, pale mucous Membranes, pyrexia, dyspnea Abdominal distension, weight loss	Blood	Negative
		Peritoneal liquid	Negative
		Feces	Negative
Domestic 4: Male, Senior, Wet-type	Diarrhea, anorexia, pale mucous Membranes, pyrexia, dyspnea Abdominal distension, weight loss	Blood	Negative
		Peritoneal liquid	Negative
		Feces	Negative
Domestic 5: Male, Kitten, Wet-type	Diarrhea, anorexia, pale mucous Membranes, pyrexia, dyspnea Abdominal distension, weight loss	Blood	Negative
		Peritoneal liquid	Positive
		Feces	Negative
Domestic 6: Male, Kitten, Dry-type	Diarrhea, anorexia, pale mucous Membranes, pyrexia, dyspnea Abdominal distension, weight loss	Blood	Negative
		Peritoneal liquid	Negative
		Feces	Negative
Persian 7: Female, Junior, Dry-type	Diarrhea, anorexia, pale mucous Membranes, pyrexia, dyspnea Abdominal distension, weight loss	Blood	Negative
		Peritoneal liquid	Negative
		Feces	Negative
Persian 8: Female, Kitten, Dry-type	Diarrhea, anorexia, pale mucous Membranes, pyrexia, Dynea Abdominal distension, weight loss	Blood Peritoneal liquid Feces	Negative Negative Negative
Persian 9: Male, Kitten, Wet-type	Diarrhea, anorexia, pale mucous Membranes, pyrexia, dyspnea Abdominal distension, weight loss	Blood	Negative
		Peritoneal liquid	Negative
		Feces	Negative
Persian 10: Male, Junior, Wet-type	Diarrhea, anorexia, pale mucous Membranes, pyrexia, dyspnea Abdominal distension, weight loss	Blood	Negative
		Peritoneal liquid	Negative
		Feces	Negative

RT-PCR=Reverse transcription-polymerase chain reaction, FIPV=Feline infectious peritonitis virus, COVID-19=Coronavirus disease 2019

**Figure-2:**
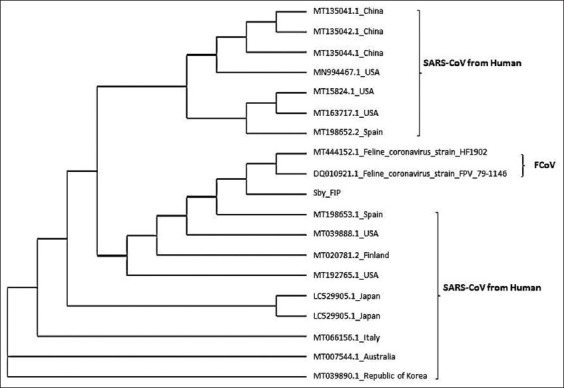
Phylogenetic tree of Sby_FIP (Surabaya Isolate), FCoV, FIPV, and SARS-CoV. FCoV=Feline coronavirus, FIPV=Feline infectious peritonitis virus, SARS-CoV=Severe acute respiratory syndrome coronavirus 2.

The PCR product was purified, after which homology and phylogenetic analyses were performed. In the homology analysis, the FIPV Surabaya isolate was shown to be 98% homologous to FCoV (MT444152) and FIPV (DQ010921) reported in GenBank. A phylogenetic tree of the FIPV Surabaya sample along with samples of FCoV, FIPV, and SARS-CoV-2 from humans (Clades A–I) was constructed. In this tree, the positive sample coded Sby_FIP was clustered together with SARS-CoV-2 Clade A (MT198653) from Spain, SARS-CoV-2 Clade A (MT192765) from the USA, SARS-CoV-2 Clade D (MT039888) from the USA, and SARS-CoV-2 Clade F (MT020781) from Finland (Figure-2).

## Discussion

In the early period after the discovery of FECV, many of its clinical signs were difficult to identify. This was similar to the case in the COVID-19 pandemic caused by SARS-CoV-2. Many questions remain about the epidemiological pathogenesis, transmission, and treatment of SARS-CoV-2. Although no reports about FECV cases during the COVID-19 pandemic in Surabaya have been published, cases of suspected FECV with FIP among domestic, Persian, and hybrid cats have increased, raising concerns among pet owners that their cats would become a source of coronavirus transmission. This anxiety about the spread of FECV and SARS-CoV-2 is often associated with these conditions initially having mild clinical signs or even being asymptomatic but potentially quickly leading to systemic disease and even death [13–15].

Infection of FCoV in cats is generally related to FECV disease and it is deemed a causative agent of mild gastrointestinal disease, being rarely encountered clinically. However, FCoV infection can deteriorate into a severe and systemic condition that leads to death, which is known as FIP and caused by FIPV [14, 15]. FIPV differs from FECV due to its ability to infect and replicate in monocytes and macrophages [14, 16] and its ability to trigger systemic inflammation. Based on FIPV’s general clinical signs, there are two types. The effusive type or wet-type can rapidly progress and often causes the accumulation of high protein exudate in the abdominal cavity or thorax. Meanwhile, the non-effusive or wet-type can affect many organ systems, but generally shows neurological and ocular signs. Non-effusive FIP generally involves a chronic disease, which is less common than effusive FIP. Feline coronavirus can also be classified into two serotypes, Type I and Type II, based on major differences in their virus protein spike, which influences receptor binding and antibody response [14, 17]. The receptor of Type II FECV is feline aminopeptidase N [14, 18], while the receptor of Type I virus has yet to be identified. Type I FECV contributes to most cases of FIP [19].

The results of this study show that the FECV sample in Surabaya has become FIP. This was proved by the RT-PCR examinations with specifically designed primers for FIPV, which resulted in one sample being positive for FIPV (10%). This sample was from a domestic male kitten aged younger than 6 months with wet-type FIP. This is in line with the findings of Norris *et al*. [20] and Riemer *et al*. [5], who reported small-scale studies supporting an association between male sex and FIP development in cats. Likewise, in COVID-19, studies by Peckham *et al*. [21] and Vahidy *et al*. [22] showed that male cats have a higher risk of more severe manifestations of COVID-19.

The anxiety of cat owners during the COVID-19 pandemic is associated with the similarities in clinical signs of cats with FIP and COVID-19, including diarrhea, anorexia, pale mucous membranes, pyrexia, dyspnea, abdominal distension, and weight loss. Wolfe and Griesemer reported similarities in the FIP clinical signs and COVID-19 [23], while Arjun *et al*. [14] stated that the symptoms and clinical signs of both FIP and COVID-19 include fever, diarrhea, weakness, anorexia, and dyspnea. However, COVID-19 also involves other specific symptoms such as fever, dry cough, weakness, shortness of breath, myalgia, anosmia (loss of the sense of smell), ageusia (loss of the sense of taste), pneumonia, and also acute respiratory distress syndrome [14, 24].

Although FIP and COVID-19 differ clinically, their closest similarity is in the extrapulmonary findings, with endothelial dysfunction as vasculitis being a pathological sign of FIP [14, 25, 26], with lesions marked by perivascular infiltration, edema, blood vessel wall degeneration, and endothelial proliferation [27]. In COVID-19 cases, it was reported that extrapulmonary signs were caused by endotheliitis mediated by virus-causing vasculitis, especially in the veins with mild involvement of arterioles [28, 29].

The phylogenetic analysis performed in this study identified a unique cluster containing the FIPV Surabaya isolate (Sby-FIP) together with FECV and SARS-CoV-2 from Clades A, D, and F, which have been reported in GenBank. This differs from a report [30] describing that the coronavirus family is classified into four genera: *Alphacoronavirus*, *Betacoronavirus*, *Gammacoronavirus*, and *Deltacoronavirus*. Coronaviruses in dogs and cats are mostly alphacoronavirus, while zoonotic coronaviruses, such as SARS-CoV, MERS-CoV, and SARS-CoV-2 infecting humans, are categorized as *Betacoronavirus*. However, this information is preliminary and still requires comprehensive support from laboratory analyses [30]. We suggest that the transmission of SARS-CoV-2 and coronavirus of other domestic animals occur, so humans should pay more attention to coronavirus infections among cats and other domestic animals.

## Conclusion

Amid the COVID-19 pandemic, FECV that developed into FIP was discovered in Surabaya in a domestic male kitten aged younger than 6 months with wet-type FIP. On homology analysis, the FIPV Surabaya isolate was found to be 98% homologous with FCoV and FIPV, while the phylogenetic analysis clustered this isolate together with SARS-CoV-2 Clade A from Spain, SARS-CoV-2 Clade A from the USA, SARS-CoV-2 Clade D from the USA, and SARS-CoV-2 Clade F from Finland.

## Authors’ Contributions

EBA: Concept and designed the study, data acquisition, and writing and critical review of the manuscript. KRI and WMY: Concept and designed the study and critical review of the manuscript. THS and NSF: Data acquisition and critical review of the manuscript. All authors have read and approved the final manuscript.
